# Supplementary material for: Structural basis of synthetic agonist activation of the nuclear receptor REV-ERB
Source: Nat Commun. 2022 Nov 21;13:7131. doi: 10.1038/s41467-022-34892-4 (PMC9681850; doi:10.1038/s41467-022-34892-4)
Supplement: Supplementary file 1 — Supplementary Information [file 41467_2022_34892_MOESM1_ESM.pdf]

Supplementary Information

Structural Basis of Synthetic Agonist Activation of the Nuclear Receptor REV-ERB

Meghan H. Murray[1,2], Aurore Cecile Valfort[3], Thomas Koelblen[3], Céline Ronin[4], Fabrice Ciesielski[4], Arindam Chatterjee[1], Giri Babu Veerakanellore[2,5], Bahaa Elgendy[2,5], John K. Walker[1], Lamees Hegazy[2,5*] and Thomas P. Burris[3*]

[1]Department of Pharmacology and Physiology, Saint Louis University School of Medicine, St. Louis, Missouri 63104, United States.

[2]Center for Clinical Pharmacology, Washington University School of Medicine, University of Health Sciences &
Pharmacy, St. Louis, MO 63110, USA

[3]University of Florida Genetics Institute, Gainesville, Florida 32610, United States

[4]NovAliX SAS, Strasbourg, France

[5]Department of Pharmaceutical and Administrative Sciences, University of Health Sciences & Pharmacy, St. Louis, MO 63110, USA

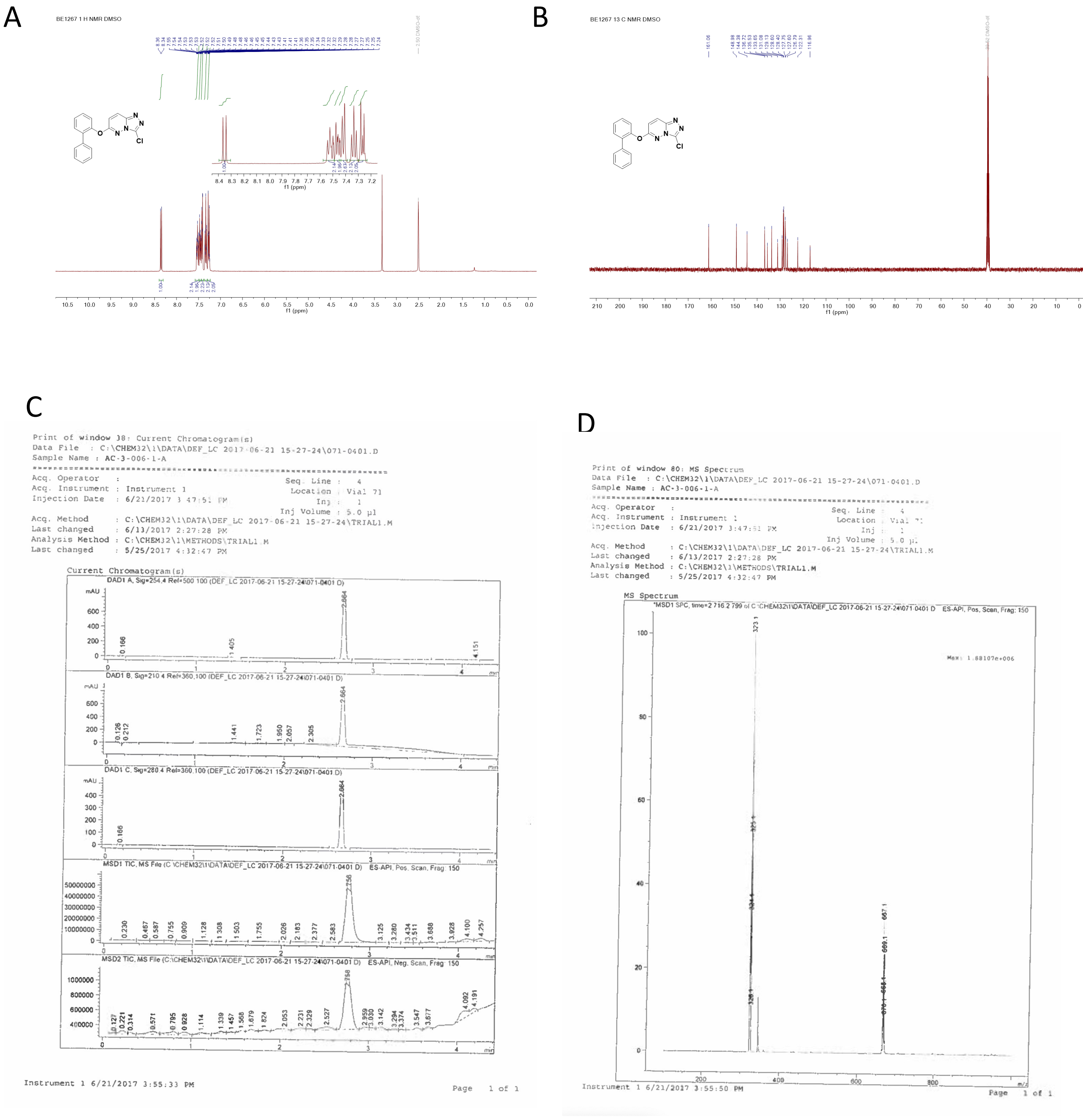

Supplementary Figure 1. Validation of STL1267 identify and purity. (**a**) ^{1}H NMR (400 MHz, DMSO-*d*6) spectrum of STL1267. (**b**)^{13}C NMR (101 MHz, DMSO-*d*6) spectrum of STL1267 (c). LC/GC data for STL1267 (d). MS data for STL1267.

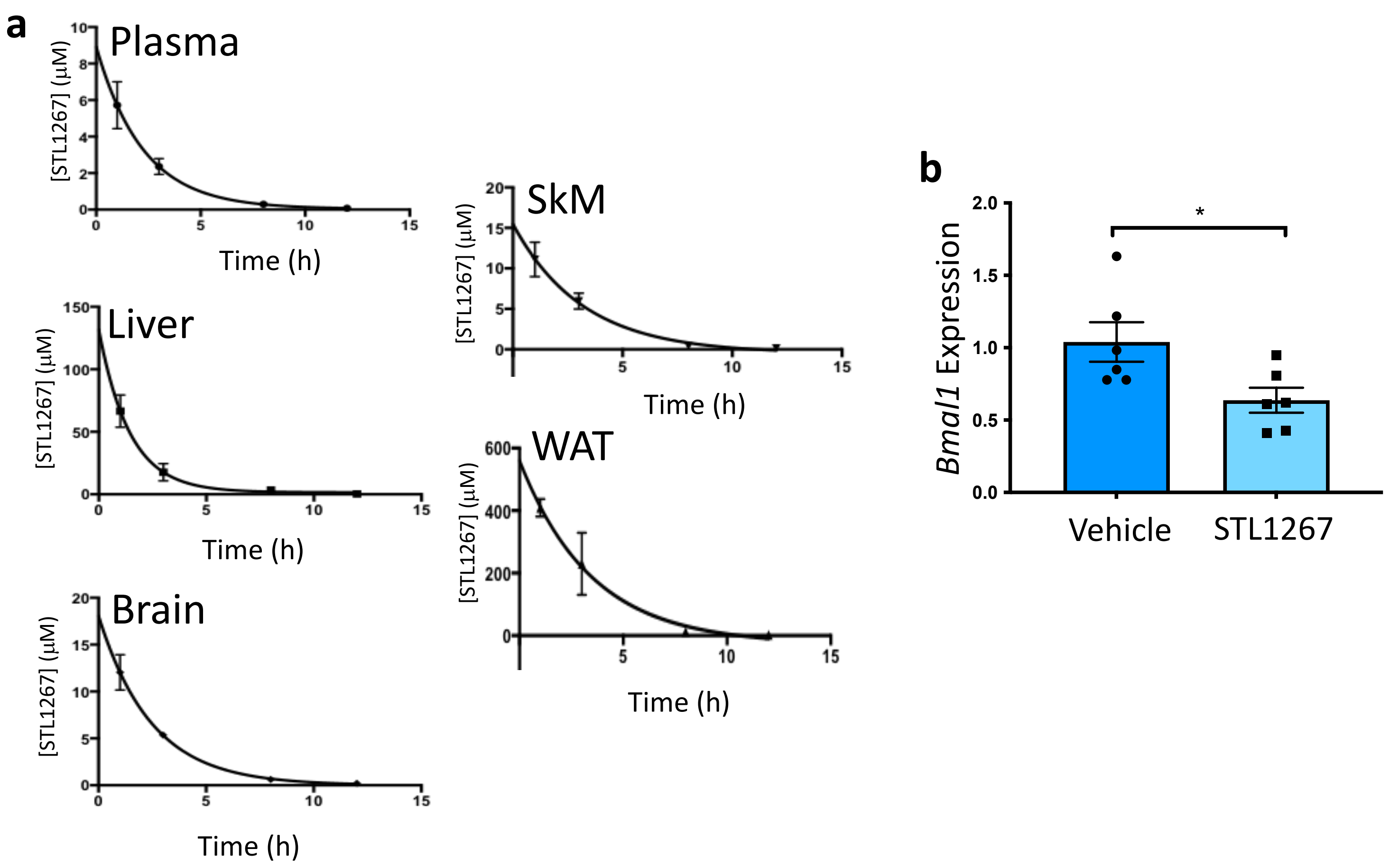

Supplementary Figure 2. In vivo exposure and activity of STL1267 when administered intraperitoneally. Mice were injected IP with 50 mg/kg STL1267 (n=6 per time point) or vehicle – 10%DMSO/12%TWEEN80/PBS (n=6 per time point). Mice were taken down 2 hours, 4 hours, 8 hours, and 12 hours after dosing. Plasma, liver, skeletal muscle, brain, and white adipose tissue was collected at each time point for mass spectrometry detecting levels of STL1267 in each tissue (**a**). At 12 hours after dosing, livers were collected for gene expression (vehicle, blue; STL1267, light blue) (**b**). *P < 0.05 by Student's t test. Data are presented as mean ± SEM

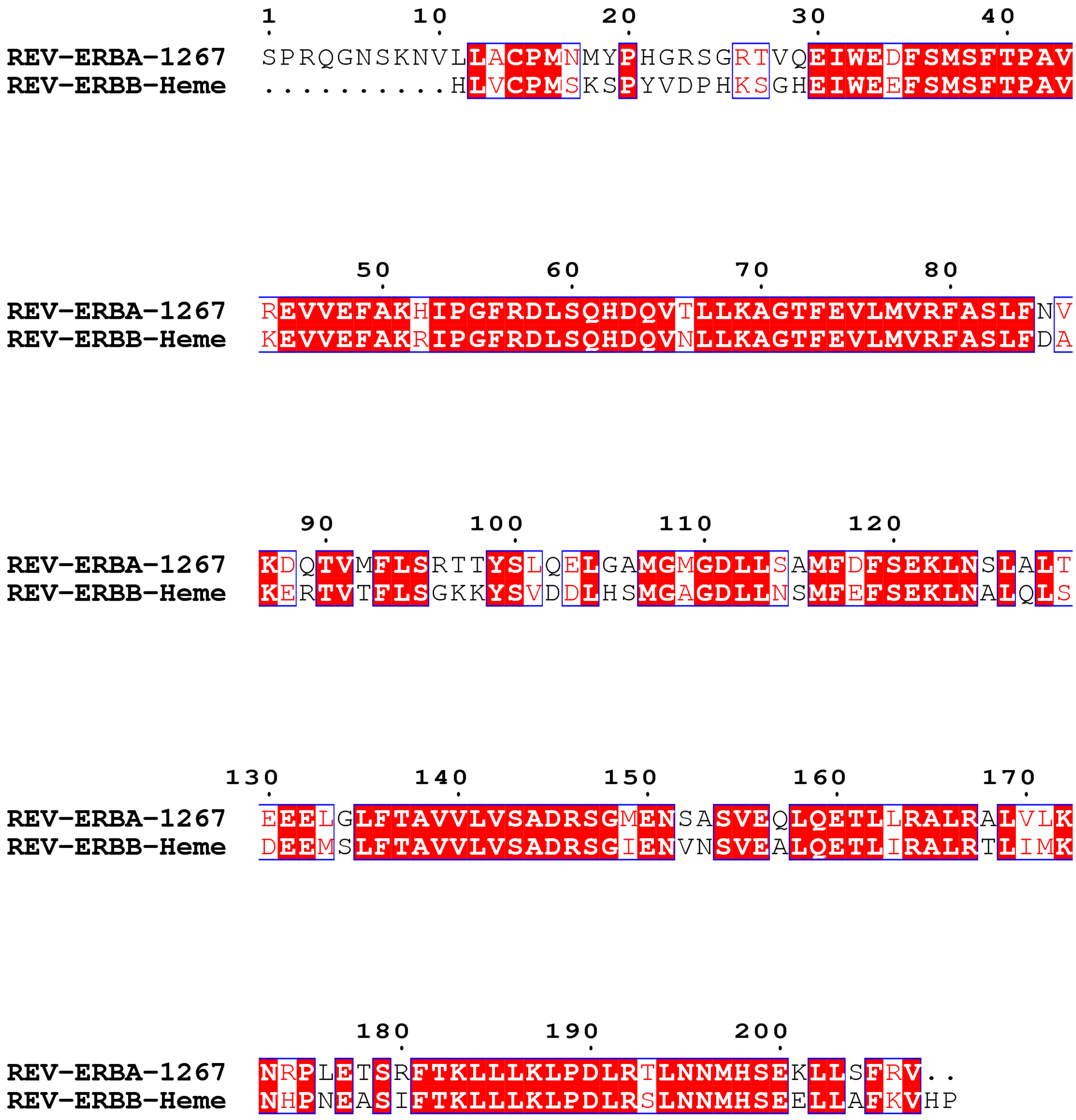

Supplementary Figure 3. Pair wise sequence alignment for REV-ERBα LBD and REV-ERBβ LBD. All residues involved in heme and 1267 are similar in both isoforms. Conserved residues between both isoforms are highlighted in red.

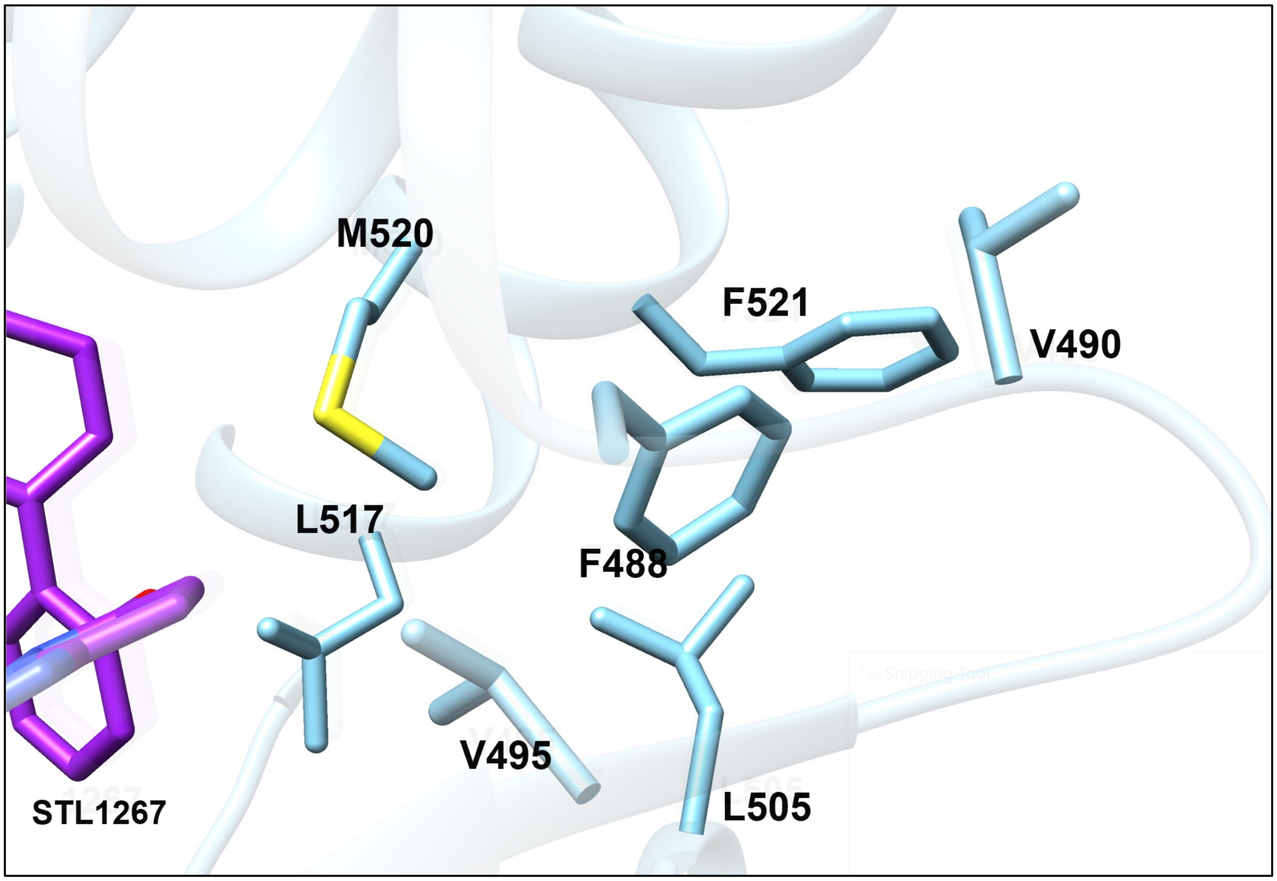

Supplementary Figure 4. **Illustration of interactions of F488 with STL1267 within the LBP of REV-ERB.** This represents a close view of molecular interactions of Phe488 after MD simulations.

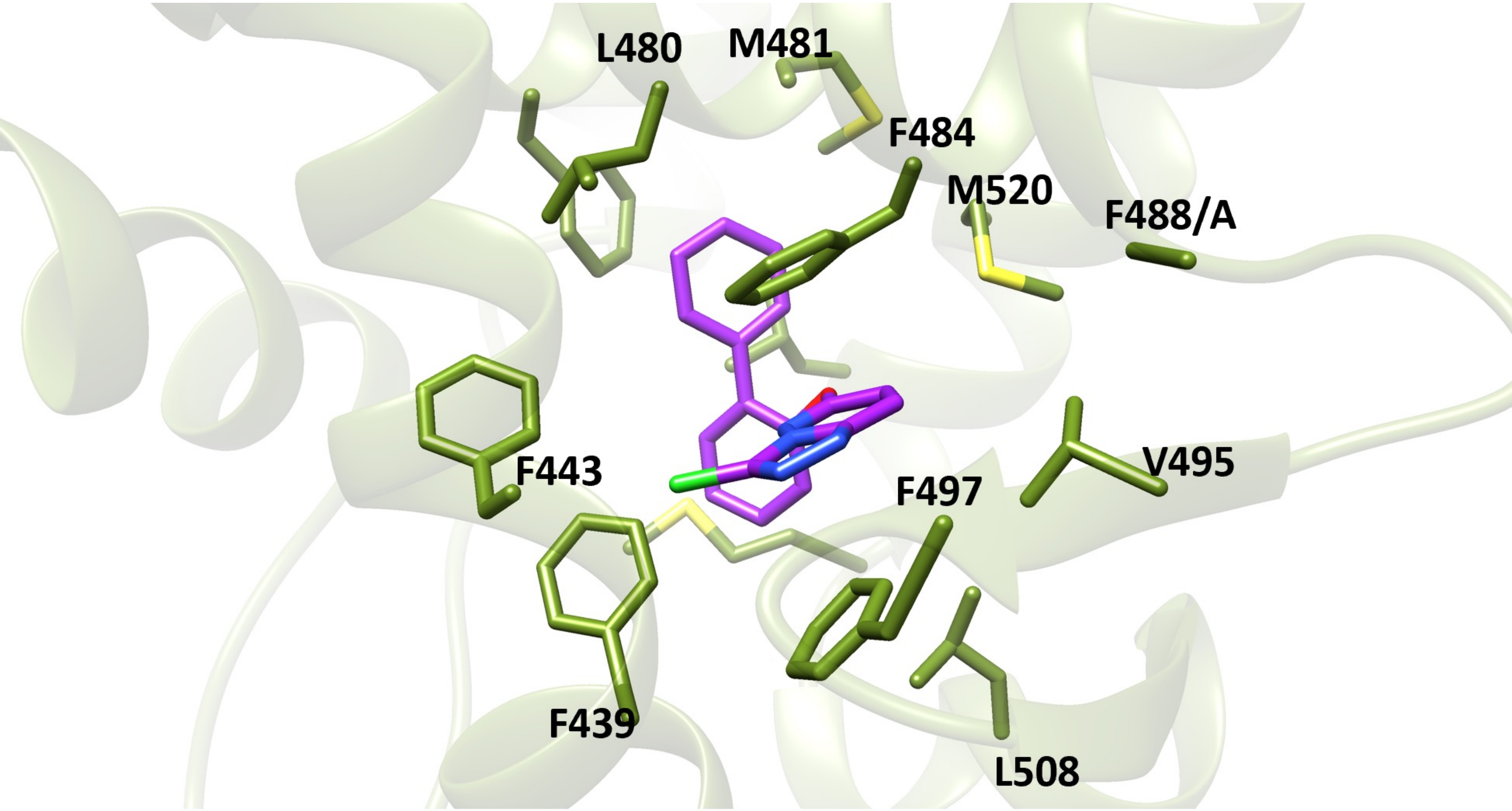

Supplementary Figure 5. Representative snapshot from the molecular dynamic simulations of the F488A mutated REV-ERBα/STL1267/NCoR ID 1. The ligand atoms are shown in purple and protein amino acids are shown in green.

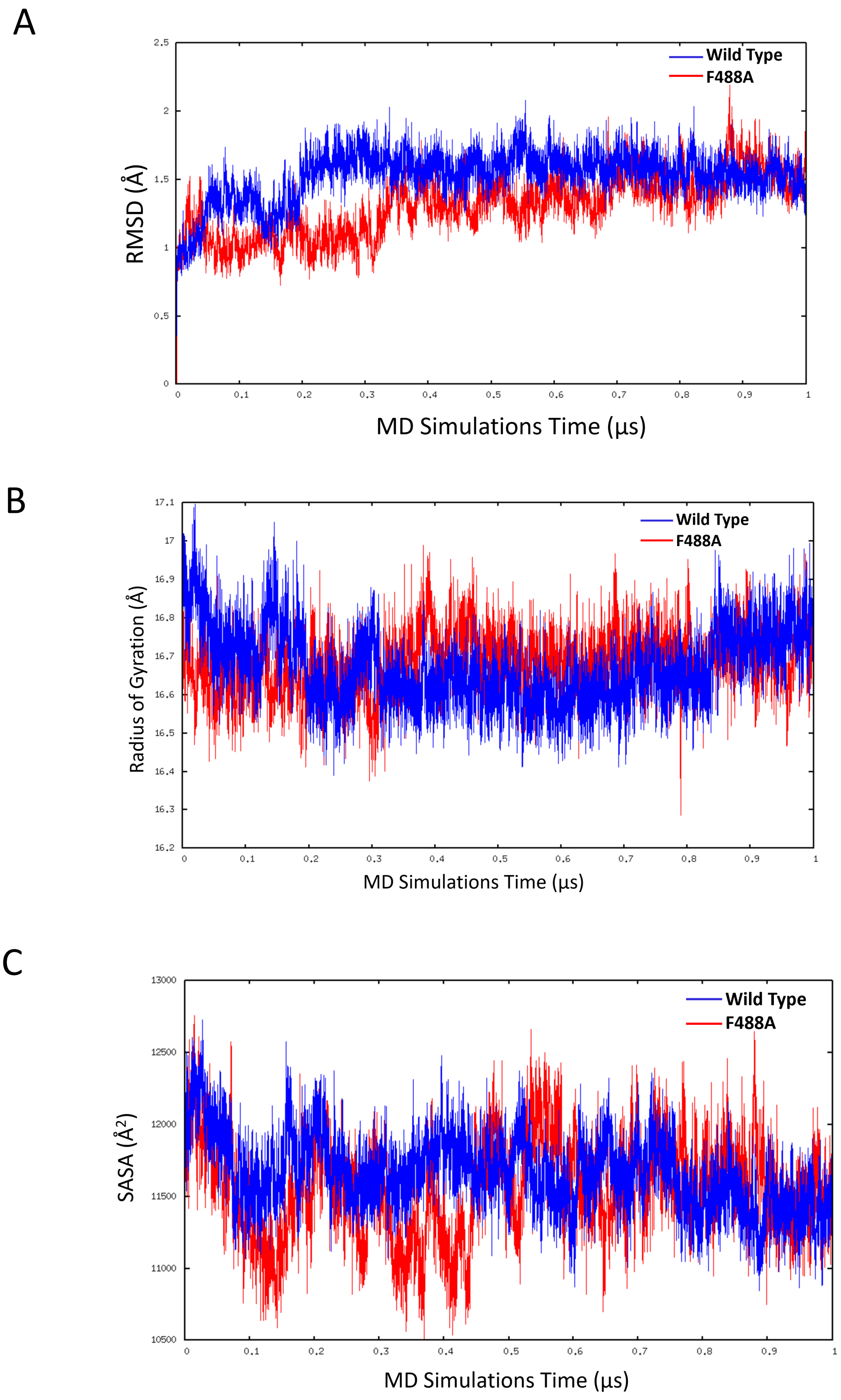

Supplementary Figure 6. Comparative analysis plots for the wild type REV-ERBα and F488A mutant REV-ERBα. (A) Root mean square deviation, RMSD, (B) Radius of gyration, Rg and (C) Solvent accessible surface area (SASA values).

A

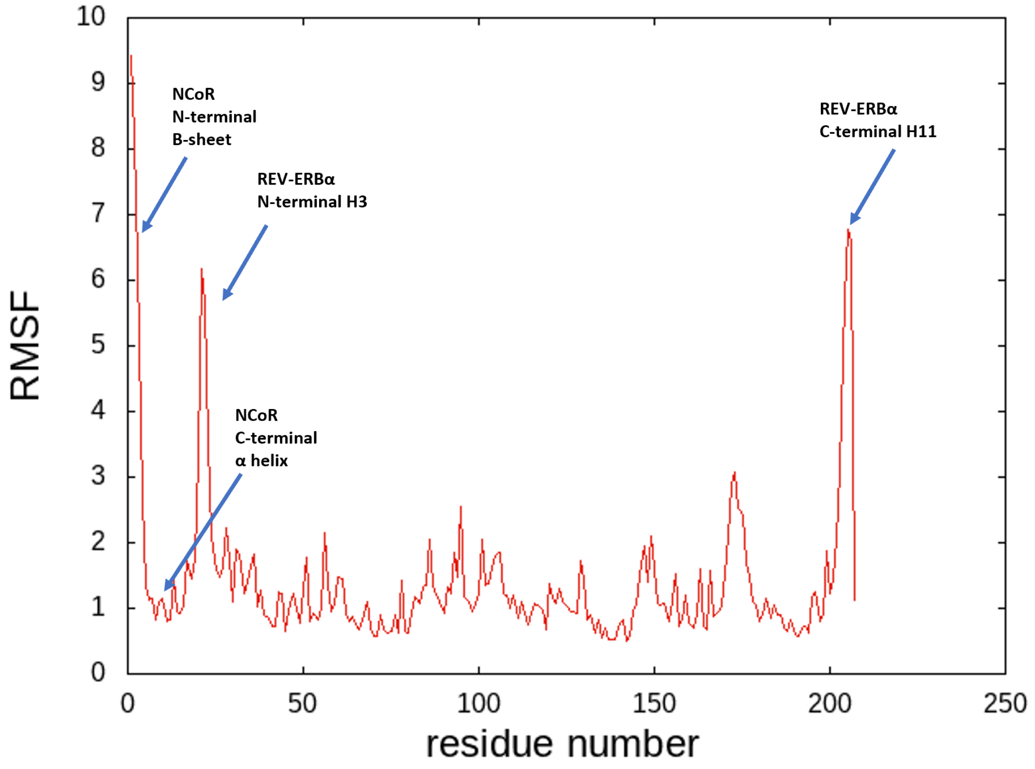

B

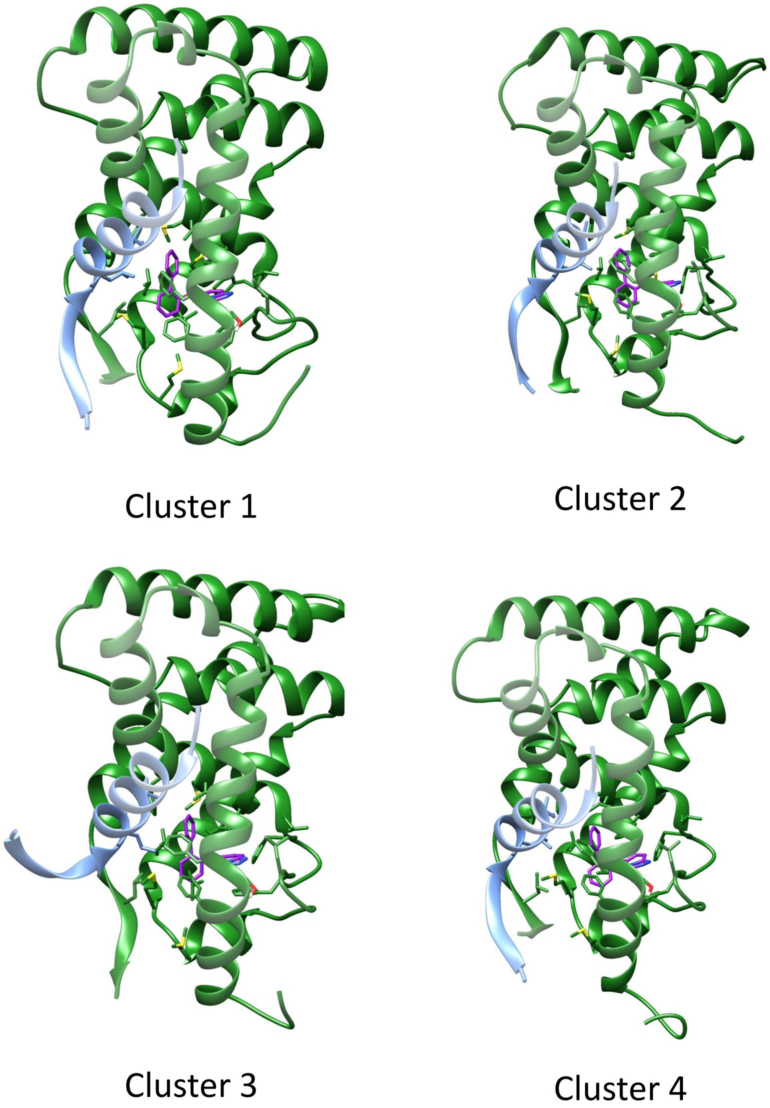

Supplementary Figure 7. **Molecular dynamic simulations indicate flexibility in the β-sheet region of the NCoR ID1 peptide bound to REV-ERBα**. A. Root mean square fluctuations (Å) calculated from the MD simulations trajectory . B. representative structures of top four populated MD simulations trajectory clusters.

PDSP			Gal NR LBD	
Receptor	RLBA Ki		Receptor	activity
5-HT1A	>10 μM		AR	n.a.
5-HT1B	>10 μM		GR	n.a.
5-HT1D	>10 μM		MR	n.a.
5-HT1E	>10 μM		PR	n.a.
5-HT2A	>10 μM		ERα	n.a.
5-HT2B	>10 μM		ERβ	n.a.
5-HT2C	>10 μM		RARa	n.a.
5-HT3	>10 μM		RXRa	n.a.
5-HT5A	>10 μM		TRa	n.a.
5-HT6	>10 μM		LXRα	n.a.
5-HT7A	>10 μM		FXR	n.a.
Alpha1A	>10 μM		VDR	n.a.
Alpha1B	>10 μM			
Alpha1D	>10 μM			
Alpha2A	>10 μM			
Alpha2B	>10 μM			
Alpha2C	>10 μM			
Beta1	>10 μM			
Beta2	>10 μM			
Beta3	>10 μM			
BZP Rat Brain Site	>10 μM			
D1	>10 μM			
D2	>10 μM			
D3	>10 μM			
D4	>10 μM			
D5	>10 μM			
DAT	>10 μM			
DOR	>10 μM			
GABAA	>10 μM			
H1	>10 μM			
H2	>10 μM			
H3	>10 μM			
H4	>10 μM			
KOR*	0.72 μM			
M1	>10 μM			
M2	>10 μM			
M3	>10 μM			
M4	>10 μM			
M5	>10 μM			
MOR	>10 μM			
NET	>10 μM			
PBR	>10 μM			
SERT*	2.1 μM			

Supplementary Table 1. Specificity of STL1267. NIMH PDSP radioligand assay specificity data for STL1267(left). Each target was saturated with a known radioligand, and 10µM STL1267 was added to the mix. If greater than 50% of the radioligand was displaced by 10µM STL1267, then secondary binding assays generating a full displacement curve was generated. Data for the secondary displacement curves is presented in Table 1.*Targets that exceeded 50% radioligand displacement by 10µM STL1267. Gal4-UAS reporter assay for nuclear receptor specificity (right). HEK293 cells transfected with Gal4-UAS luciferase and Gal4 DBD- NR treated with 10 μM 1267. n.a. indicates no detectable activity.

Supplementary Table 2. Primers for RT-qPCR

Target	Forward Sequence	Reverse Sequence
Bmal1	GGACTTCGCCTCTACCTGTTC	ACCCGTATTTCCCCGTTC
Mt-Nd1	GTTGGTCCATACGGCATTTT	TGGGTGTGGTATTGGTAGGG
Mt-Co1	ACTATACTACTAACAGACCG	GGTTCTTTTTTTCCGGAGTA
VLCAD	CTCAGTGAAGAACAGGCACAA	CTTGGCAGGGTCATTCACTT
LCAD	ATCTTTTCCTCGGAGCATGA	TTTCTCTGCGATGTTGATGC
SCAD	TGACTTTGCCGAGAAGGAGT	ACTCAGCTCCTCTGGCACAT
Lkb1	AGCAGTCTTGACGCAGACCT	CAAAGTCACCAAGTGCTCCA
Sirt1	TCTCCTGTGGGATTCCTGAC	ACACAGAGACGGCTGGAACT
Nampt	TCCGGCCCGAGATGAAT	GTGGGTATTGTTTATAGTGAGTAACC TTGT
PPARGCoA1	GGAGCTCCAAGACTCTAGACA	CCAAAGTCTCTCTCAGGTAGC
Ppia	GCATACGGGTCCTGGCATCTTGTCC	ATGGTGATCTTCTTGCTGGTCTTGC

Supplementary Table 3. Data collection and refinement statistics for REV-ERBα bound with STL1267.

	Rev-erbα/STL1267
**Data collection**	
Space group	R32
Cell dimensions	
a, *b*, *c* (Å)	115.07, 115.07, 107.32
α, β, γ (°)	90, 90, 120
Resolution (Å)	73.03-2.50 (2.73-2.50)
Rpim (%)	2.6 (51.7)
$I / \sigma I$	18.4 (1.4)
Completeness (%)	85.5 (32.6)
Redundancy	8.1 (8.1)
**Refinement**	
Resolution (Å)	73.03-2.50
No. reflections	7248
R_{work} / R_{free}	20.52 / 23.95
No. atoms	
Protein	1601
Peptide	160
Ligand	23
Water	26
B-factors	
Protein	77.61
Peptide	84.24
Ligand/ion	62.74
Water	71.81
R.m.s. deviations	
Bond lengths (Å)	0.008
Bond angles (°)	0.930

Supplementary Table 4. **Contribution of the binding energy components to the total binding free energy, ΔG (Kcal/mol)**. ΔH corresponds to the favorable affinity contribution, while ΔS is the entropy and reflects the decrease in conformational freedom in the protein ligand complex.

REV-ERB/STL1267/NCOID1	ΔH (Kcal/mol)	TΔS (Kcal/mol)	ΔG = ΔH - TΔS (Kcal/mol)
**Wild type**	-42.3	-21.5	-20.8
**Mutant**	-43.4	-19.0	-24.4

Supplementary Methods

SR9009. SR9009[1] was synthesized by reductive amination of 5-nitro-2-thiophenecarboxaldehyde with 4-chlorobenzylamine, and sodium triacetoxyborohydride yielded the secondary amine. A second reductive amination with 1-Boc-pyrrolidine-3-carboxaldehyde yielded the tertiary amine. This compound was treated with trifluoroacetic acid to remove the Boc-protecting group, and then reacted with either ethyl chloroformate to give the desired products[1].

Synthesis of 6-([1,1'-biphenyl]-2-yloxy)-3-chloro-[1,2,4]triazolo[4,3-b]pyridazine (STL1267):

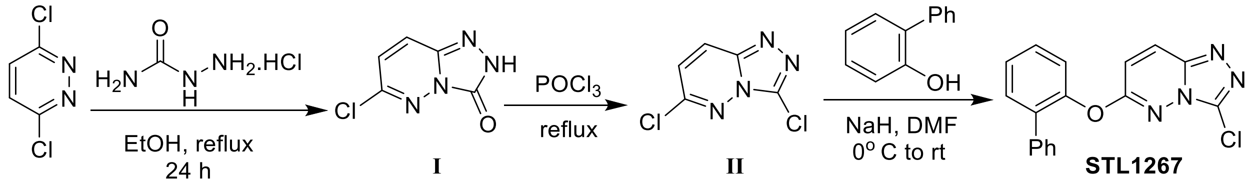

Step 1: Synthesis of 6-chloro-[1,2,4]triazolo[4,3-b]pyridazin-3(2H)-one (I) – To a 500 mL round bottom flask, 3,6-dichloropyridazine (10 g, 1 equiv.) was added and dissolved in ethanol (100 mL). Semicarbazide hydrochloride (15 g, 2.004 equiv.) was dissolved in H_2O (180 mL) and was added to the reaction mixture dropwise. The reaction was heated under reflux for 24 hr. After complete conversion, the reaction mixture was cooled to room temperature (rt) and stirred for 3 d. The precipitated solid was filtered, washed and dried under vacuum to give the desired product in a pure form for the next step (Yield = 37.3% (4.24 g)).

Step 2: Synthesis of 3,6-dichloro-[1,2,4]triazolo[4,3-b]pyridazine (II)[2] – In a 500 mL RB flask, compound I (4.24 g) was dissolved in $POCl_3$ (20 equiv.) and heated to reflux for 48 h. The progress of reaction was monitored by TLC and LC-MS. After complete conversion, the whole reaction was quenched with by adding the crude reaction mixture dropwise to a mixture of crushed ice and cold water. The crude product was extracted twice with ethyl acetate (2 x 100 mL). The combined organic layers were dried over anhydrous Na_2SO_4 and the solvent was evaporated under reduced pressure. The crude product was obtained as a light greenish white solid and was crystallized from ethyl acetate (Yield: 78.7% (3.7 g)).

Step 3: Synthesis of 6-([1,1'-biphenyl]-2-yloxy)-3-chloro-[1,2,4]triazolo[4,3-b]pyridazine (STL1267) – To a 8 mL vial, [1,1'-biphenyl]-2-ol (100 mg, 1 equiv.) was dissolved in DMF (2 mL) and cooled to 0 °C. Sodium hydride (28.2 mg, 2 equiv.) was added and the reaction mixture was stirred for 3-5 minutes. To the above reaction mixture, compound II (111 mg, 1 equiv.) was added, and the reaction was stirred for 1 h. Reaction progress was monitored by TLC. After reaction completion, the mixture was quenched with crushed ice and the crude product was extracted with ethyl acetate (2 x 20 mL). The combined organic layers were dried over anhydrous sodium sulphate and purified by flash column chromatography. White solid (113.6 mg, 0.352 mmol, 59.9%), R_f = 0.38 (ethyl acetate/hexanes = 1:1 (v/v)); m.p. 164-165 °C. ^1H NMR (400 MHz, DMSO-*d*$_6$) δ 8.35 (d, *J* = 9.8 Hz, 1H), 7.58-7.49 (m, 2H), 7.49-7.44 (m, 2H), 7.42 (dd, *J* = 8.0, 1.4 Hz, 2H), 7.33 (dd, *J* = 8.3, 6.7 Hz, 2H), 7.30-7.23 (m, 2H). ^{13}C NMR (100 MHz, DMSO-*d*$_6$) δ 161.06, 148.98, 144.38, 136.72, 135.53, 133.65, 131.08, 129.13, 128.60, 128.40, 127.75, 127.60, 126.79, 122.31, 116.96. HRMS (EI) m/z: [M+ Na]+ calcd for $C_{17}H_{11}ClN_4ONa$, 345.051360; found, 345.051357. HPLC: Ascentis express peptide ES C-18 column, OD 3cm X 4.6cm, 6min flow rate 1 mL/min; gradient = 95/5 → 5/95 CH3CN-H2O). The retention time for STL1267 was 2.66 min.

Supplementary References

(1) Solt, L. A.; Wang, Y.; Banerjee, S.; Hughes, T.; Kojetin, D. J.; Lundasen, T.; Shin, Y.; Liu, J.; Cameron, M. D.; Noel, R.; et al. Regulation of Circadian Behavior and Metabolism by Synthetic REV-ERB Agonists. *Nature* **2012**, *485*, 62-68.
(2) Yang, Y.; Zhang, Y.; Yang, L.; Zhao, L.; Si, L.; Zhang, H.; Liu, Q.; Zhou, J. Discovery of imidazopyridine derivatives as novel c-Met kinase inhibitors: Synthesis, SAR study, and biological activity. *Bioorg Chem* **2017**, *70*, 126-132. DOI: 10.1016/j.bioorg.2016.12.002.
