# Supplementary material for: Structural basis of synthetic agonist activation of the nuclear receptor REV-ERB
Source: Nat Commun. 2022 Nov 21;13:7131. doi: 10.1038/s41467-022-34892-4 (PMC9681850; doi:10.1038/s41467-022-34892-4)
Supplement: Supplementary file 3 — Description of Additional Supplementary Files [file 41467_2022_34892_MOESM3_ESM.pdf]

Description of Additional Supplementary Files

File Name: Supplementary Data 1
Description: Initial configuration for the REVERBα/STL1267/NCoR ID1 molecular simulations

File Name: Supplementary Data 2
Description: Final configuration for the REVERBα/STL1267/NCoR ID1 molecular simulations

File Name: Supplementary Movie 1
Description: e. Movie depicting the molecular dynamic simulation REVERBα/STL1267/NCoR ID1 complex showing increased flexibility of the β sheet regions of the amino terminus of NCoR ID1 and the carboxy-terminus of REV-ERBα.
