# Supplementary material for: Structural basis of synthetic agonist activation of the nuclear receptor REV-ERB
Source: Nat Commun. 2022 Nov 21;13:7131. doi: 10.1038/s41467-022-34892-4 (PMC9681850; doi:10.1038/s41467-022-34892-4)
Supplement: Supplementary file 7 — Reporting Summary [file 41467_2022_34892_MOESM7_ESM.pdf]

nature portfolio

Corresponding author(s): Thomas Burris and Lamees Hegazy

Last updated by author(s): Nov 7, 2022

Reporting Summary

Nature Portfolio wishes to improve the reproducibility of the work that we publish. This form provides structure for consistency and transparency in reporting. For further information on Nature Portfolio policies, see our Editorial Policies and the Editorial Policy Checklist.

Statistics

For all statistical analyses, confirm that the following items are present in the figure legend, table legend, main text, or Methods section.

n/a	Confirmed	
☐	☒	The exact sample size (*n*) for each experimental group/condition, given as a discrete number and unit of measurement
☐	☒	A statement on whether measurements were taken from distinct samples or whether the same sample was measured repeatedly
☐	☒	The statistical test(s) used AND whether they are one- or two-sided<br>*Only common tests should be described solely by name; describe more complex techniques in the Methods section.*
☒	☐	A description of all covariates tested
☒	☐	A description of any assumptions or corrections, such as tests of normality and adjustment for multiple comparisons
☒	☐	A full description of the statistical parameters including central tendency (e.g. means) or other basic estimates (e.g. regression coefficient) AND variation (e.g. standard deviation) or associated estimates of uncertainty (e.g. confidence intervals)
☐	☒	For null hypothesis testing, the test statistic (e.g. *F*, *t*, *r*) with confidence intervals, effect sizes, degrees of freedom and *P* value noted<br>*Give P values as exact values whenever suitable.*
☒	☐	For Bayesian analysis, information on the choice of priors and Markov chain Monte Carlo settings
☒	☐	For hierarchical and complex designs, identification of the appropriate level for tests and full reporting of outcomes
☒	☐	Estimates of effect sizes (e.g. Cohen's *d*, Pearson's *r*), indicating how they were calculated

Our web collection on statistics for biologists contains articles on many of the points above.

Software and code

Policy information about availability of computer code

Data collection	SCIEX OX 3.0
Data analysis	GraphPad Prism 9.0, Amber 18, UCSF Chimera 1.15, Schrodinger Maestro 2021 release, Gnuplot 4.6, Autoprox/Staranisio v3, Buster release 03.02.2022, Grade 1.2.19, MolProbity 4.3

For manuscripts utilizing custom algorithms or software that are central to the research but not yet described in published literature, software must be made available to editors and reviewers. We strongly encourage code deposition in a community repository (e.g. GitHub). See the Nature Portfolio guidelines for submitting code & software for further information.

Data

Policy information about availability of data

All manuscripts must include a data availability statement. This statement should provide the following information, where applicable:

- Accession codes, unique identifiers, or web links for publicly available datasets
- A description of any restrictions on data availability
- For clinical datasets or third party data, please ensure that the statement adheres to our policy

Data availability statement: All macromolecular structure data generated in this study has been deposited to the Protein Data BAnk under the following accession number: PDB ID:8D8I. All the data included in the manuscript and Supplementary Information are available from the corresponding author upon request.

Human research participants

Policy information about studies involving human research participants and Sex and Gender in Research.

Reporting on sex and gender	no human research participants
Population characteristics	no human research participants
Recruitment	no human research participants
Ethics oversight	no human research participants

Note that full information on the approval of the study protocol must also be provided in the manuscript.

Field-specific reporting

Please select the one below that is the best fit for your research. If you are not sure, read the appropriate sections before making your selection.

[x] Life sciences [ ] Behavioural & social sciences [ ] Ecological, evolutionary & environmental sciences

For a reference copy of the document with all sections, see nature.com/documents/nr-reporting-summary-flat.pdf

Life sciences study design

All studies must disclose on these points even when the disclosure is negative.

Sample size	Sample sizes were determined based on the basis of cohort size and previously published effect size (Solt et al. Nature 2012)
Data exclusions	No samples were excluded
Replication	Animal experiments were performed once with n=3-4 and biochemical experiments were performed 2 or 3 times with 3-6 replicates per point.
Randomization	For mouse studies, animals were randomly allocated into an experimental group at the beginning of experimentation.
Blinding	Researchers and personnel involved in drug administration were not blinded. Injection volumes and tissue collection were standardized in order to prevent bias. RNA/QPCR samples were processed using standardized methods to prevent bias.

Reporting for specific materials, systems and methods

We require information from authors about some types of materials, experimental systems and methods used in many studies. Here, indicate whether each material, system or method listed is relevant to your study. If you are not sure if a list item applies to your research, read the appropriate section before selecting a response.

Materials & experimental systems

n/a	Involved in the study	
[x]	[ ]	Antibodies
[ ]	[x]	Eukaryotic cell lines
[x]	[ ]	Palaeontology and archaeology
[ ]	[x]	Animals and other organisms
[x]	[ ]	Clinical data
[x]	[ ]	Dual use research of concern

Methods

n/a	Involved in the study	
[x]	[ ]	ChIP-seq
[x]	[ ]	Flow cytometry
[x]	[ ]	MRI-based neuroimaging

Eukaryotic cell lines

Policy information about cell lines and Sex and Gender in Research

Cell line source(s)	All cell lines were obtained from the ATCC (C2C12, HEK293 and HepG2)
Authentication	Cell lines were obtained from the ATCC and regularly authenticated by STR profiling
Mycoplasma contamination	All cell lines were tested frequently for mycoplasma contamination and only cells that were free from contamination were used.
Commonly misidentified lines (See ICLAC register)	none utilized

Animals and other research organisms

Policy information about studies involving animals; ARRIVE guidelines recommended for reporting animal research, and Sex and Gender in Research

Laboratory animals	C57Bl6 male mice were used (6-8 weeks of age)
Wild animals	No wild animals were used
Reporting on sex	Male mice were used to avoid variation in gene expression associated with the estrus cycle.
Field-collected samples	No field collected samples.
Ethics oversight	All in vivo studies were performed using the ARRIVE guidelines and approved by the

Note that full information on the approval of the study protocol must also be provided in the manuscript.
